# Anhydrobiotic chironomid larval motion-based multi-sensing microdevice for the exploration of survivable locations

**DOI:** 10.1016/j.isci.2022.104639

**Published:** 2022-07-20

**Authors:** Yo Tanaka, Doudou Ma, Satoshi Amaya, Yusufu Aishan, Yigang Shen, Shun-ichi Funano, Tao Tang, Yoichiroh Hosokawa, Oleg Gusev, Takashi Okuda, Takahiro Kikawada, Yaxiaer Yalikun

**Affiliations:** 1 Center for Biosystems Dynamics Research (BDR), RIKEN, 1-3 Yamadaoka, Suita, Osaka 565-0871, Japan; 2 Graduate School of Frontier Biosciences, Osaka University, 1-3 Yamadaoka, Suita, Osaka 565-0871, Japan; 3 Graduate School of Nara Institute of Science and Technology, 8916-5 Takayamacho, Ikoma, Nara 630-0192, Japan; 4 RIKEN Cluster for Science, Technology and Innovation Hub (RCSTI), RIKEN, 1-7-22 Suehiro-cho, Tsurumi-ku, Yokohama, Kanagawa 230-0045, Japan; 5 Institute of Agrobiological Sciences, NARO, 1-2 Owashi, Tsukuba, Ibaraki 305-8634, Japan

**Keywords:** Sensor, Animal physiology, Nanotechnology, Space sciences

## Abstract

African chironomid (*Polypedilum vanderplanki*) larvae can suspend their metabolism by undergoing severe desiccation and then resume this activity by simple rehydration. We present a microdevice using interdigital comb electrodes to detect the larval motion using the natural surface charge of the living larvae in water. The larvae were most active 2 h after soaking them in water at 30°C; they exhibited motions with 2 Hz frequency. This was comparable to the signal obtained from the microdevice via fast Fourier transform (FFT) processing. The amplitude of the voltage and current were 0.11 mV and 730 nA, respectively. They would be enough to be detected by a low power consumption microcomputer. Temperature and pH sensing were demonstrated by detecting the vital motions of the revived larvae under different conditions. This multi-functional biosensor will be a useful microdevice to search for survivable locations under extreme environmental conditions like those on other planets.

## Highlights

• A biosensor using anhydrobiotic chironomid larva was created
• Interdigital comb electrodes were fabricated to detect the larvae motion
• Multi-parameters such as temperature or pH can be detected
• The device can search survivable locations under extreme environmental conditions

## Graphical abstract

**Figure:**
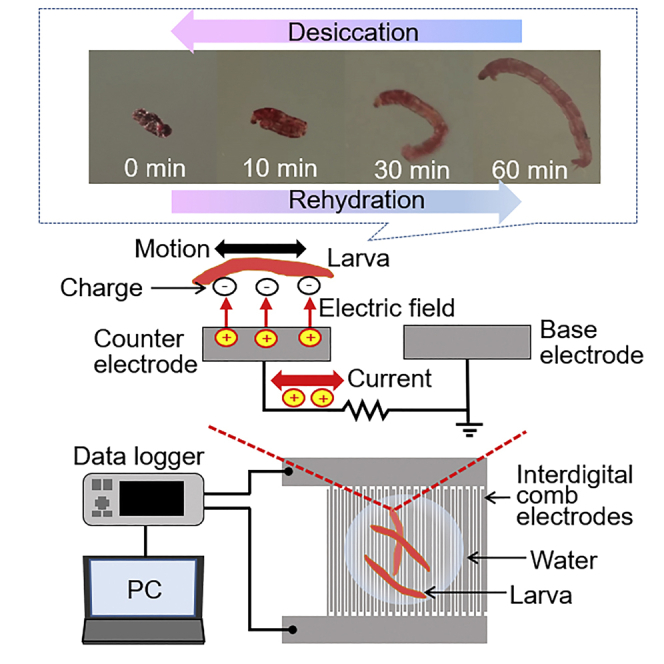

## Introduction

Explorations of Earth’s solar system and of distant galaxies are important to answer fundamental scientific questions, including the formation of the universe, the origin of Earth, the evolution of life, and the existence of life beyond Earth (Gao and Chien, 2017). Cosmic resources offer a broad area for development and they include a high-vacuum resource, a high-cleanliness resource, a microgravity resource, a solar energy resource, an ultra-low temperature resource, and rich mineral resources from the moon and other planetary objects. Within this context of obtaining basic scientific knowledge and developing cosmic resources, seeking and creating survivable environments is indispensable for the evolution of humans to alleviate pressures on Earth which are caused by population growth and energy depletion, and explorations of other planetary surfaces have received attention as they may lead to the discovery of new energy resources and living environments (Grotzinger et al., 2012). For these, the assessment of the effects of the environmental conditions on survivability is necessary, and a variety of sensors are needed to investigate various parameters including the electromagnetic field (Díaz-Michelena, 2009), chemical toxicity of the environment, its water and air availability, its temperature, and its availability of nutrients (Sanz et al., 2013). Moreover, there is a possibility that unknown parameters affect biological adaptation.

**Figure 1:**
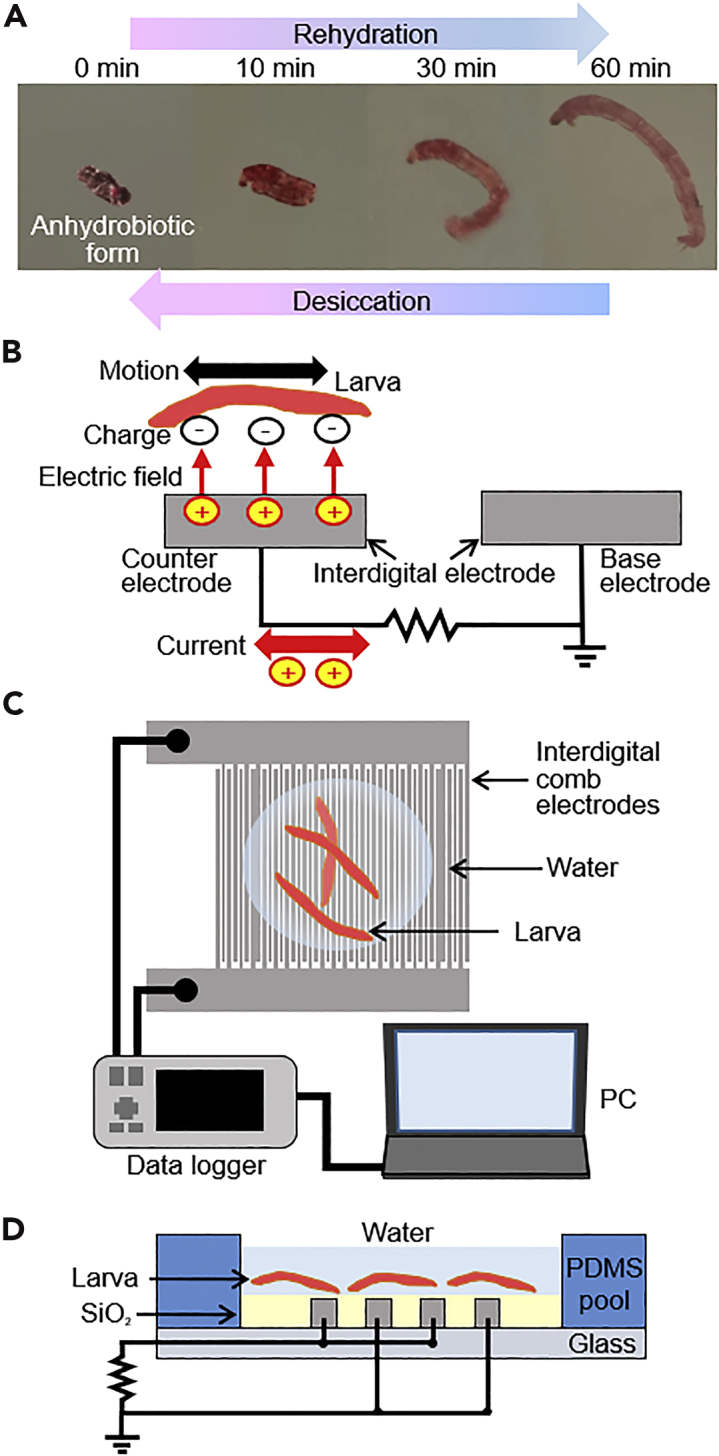
Design and principle of the microdevice (A) Photos showing anhydrobiosis of a larva. (B) Principle of the vibration-based power generation device that uses the motion of the larva. (C) Set-up with the microdevice for measurements in this study. (D) Cross-sectional view of the microdevice with a PDMS pool for the confinement of larvae and water.

A simple method to overcome the need to use numerous sensors is to bring organisms to the actual site and see whether they can survive there. A life-mechanical fusion sensor is a convenient and reliable detection tool that directly transports organisms to the surface of a planet for the assessment of the environmental conditions through the activities of the organisms. However, there are few organisms that can survive in extreme environments, and therefore long-term monitoring is difficult. Here, we focused on the larva of the sleeping chironomid, *Polypedilum vanderplanki*. This species lives in puddles on granite rocks in the semi-arid region of sub-Saharan Africa, and it has a very distinctive ability called “anhydrobiosis.” As the surrounding water evaporates, the larvae gradually dry out, and eventually, their body water content falls to less than 3% of their body weight and all metabolism ceases (Cornette and Kikawada, 2011). In this state, the larvae can endure extreme temperatures of −270 to 90°C (Hinton, 1960; Watanabe et al., 2002; Sakurai et al., 2008), and irradiation by γ-rays over 5000 Gy in absorbed dose (Watanabe et al., 2006a) or irradiation by ion beams (Watanabe et al., 2006b). Upon contact with water, the larvae rapidly absorb water and return to an active state within 1 h (Figure 1A). This unique ability is attributed primarily to anhydrobiosis-related gene products that are activated by desiccation-specific regulatory mechanisms and to the vitrification of trehalose, which is accumulated in the desiccated larvae (Ryabova et al., 2020; Mazin et al., 2018). Surprisingly, the larvae could be recovered even 17 years after their desiccation (Adams, 1983). Owing to such long-term endurance to extreme environments, they were tested by being kept in outer space on the international space station (ISS) module for 2.5 years before being successfully recovered (Novikova et al., 2011). Our concept is to exploit this unique property of a dried larva as a sensing organism. When encountering a watery and survivable environment, the dried larva gradually recovers and begins to move. This motion is the total expression of various sensing performances of the larva. By using a wireless communication device with the larva, this information about restored movement activity can be sent immediately.

However, the larva is small (about 2 mm in length and 0.2 mm in diameter) and its motion is small as well, even though it is far larger than other similar and better-known anhydrobiotic organisms: Tardigrada (Jönsson et al., 2016), algae (Wang et al., 2021), or bacteria (Safary et al., 2002) which also can endure extreme environments. Therefore, to realize this concept, a highly sensitive detection method is required. For this purpose, it is natural to use a high-resolution camera to monitor the small motion. However, a camera requires a continuous power source, and a large battery is required for long-term monitoring, but that is not realistic considering the conditions for use. Therefore, we focused on an energy harvesting technology based on microelectromechanical systems (MEMS) technology which can detect small vibrations (Beeby et al., 2006; Saadon et al., 2011). By exploiting the vibration-based electric power generation, power-free detection of motion becomes possible. By constructing a microdevice to start monitoring and wireless communication of the electrical signal, we will be able to realize long-term monitoring with an as-small-as-possible power requirement.

In this article, we demonstrated a microdevice for survivable space exploration that uses vibration-based electric power generation exploiting the property of the anhydrobiotic chironomid larva motion. First, the motion of the chironomid larva was investigated using image data. Second, the power generation performance was estimated by simulation based on the motion and the electrical property of the larva. Third, the vibration-based power generation device was constructed based on the results, and its function was verified in two ways: by a spontaneous motion of the larva and by use of a constant vibration machine. Finally, the dependency of the data on temperature and pH was analyzed to show the possibility of using the microdevice as a multi-biosensor.

The principle of the microdevice is shown in Figure 1B. In vibration-based electrostatically energy-harvesting devices (Suzuki and Tai, 2006; Khan and Qadir, 2016; Zhang et al., 2018), an electric current is generated by the potential difference when the larva actuates the microdevice with the interdigital comb electrodes as microorganisms generally have a negative surface charge in water (Huang et al., 2018). The mechanism of the power generation is briefly as follows (Suzuki, 2011). The device has “base electrode” and “counter electrode.” “Base electrode” is the one connected to earth. Charges with the opposite sign are induced on the counter electrode by the electrostatic field of the larvae, and the amount of the induced charges changes with the overlapping area between the charged material and the counter electrode, generating an alternating current in the external circuit. The potential of the “base electrode” is always 0 V. On the other hand, “counter electrode” has a positive charge because of the permanent negative charge of the larvae surface in water. Because individual differences among larval motions are large, the average motion of the larvae group must be analyzed. Therefore, we designed the MEMS-based interdigital comb electrodes to cover a large area (about 1 × 1 cm) to sense the motion of about 10 larvae simultaneously, and a polydimethylsiloxane (PDMS) pool was used to confine the larvae as shown in Figures 1C and 1D.

## Results

### Investigation of the rough motion property of a group of chironomid larvae

**Figure 2:**
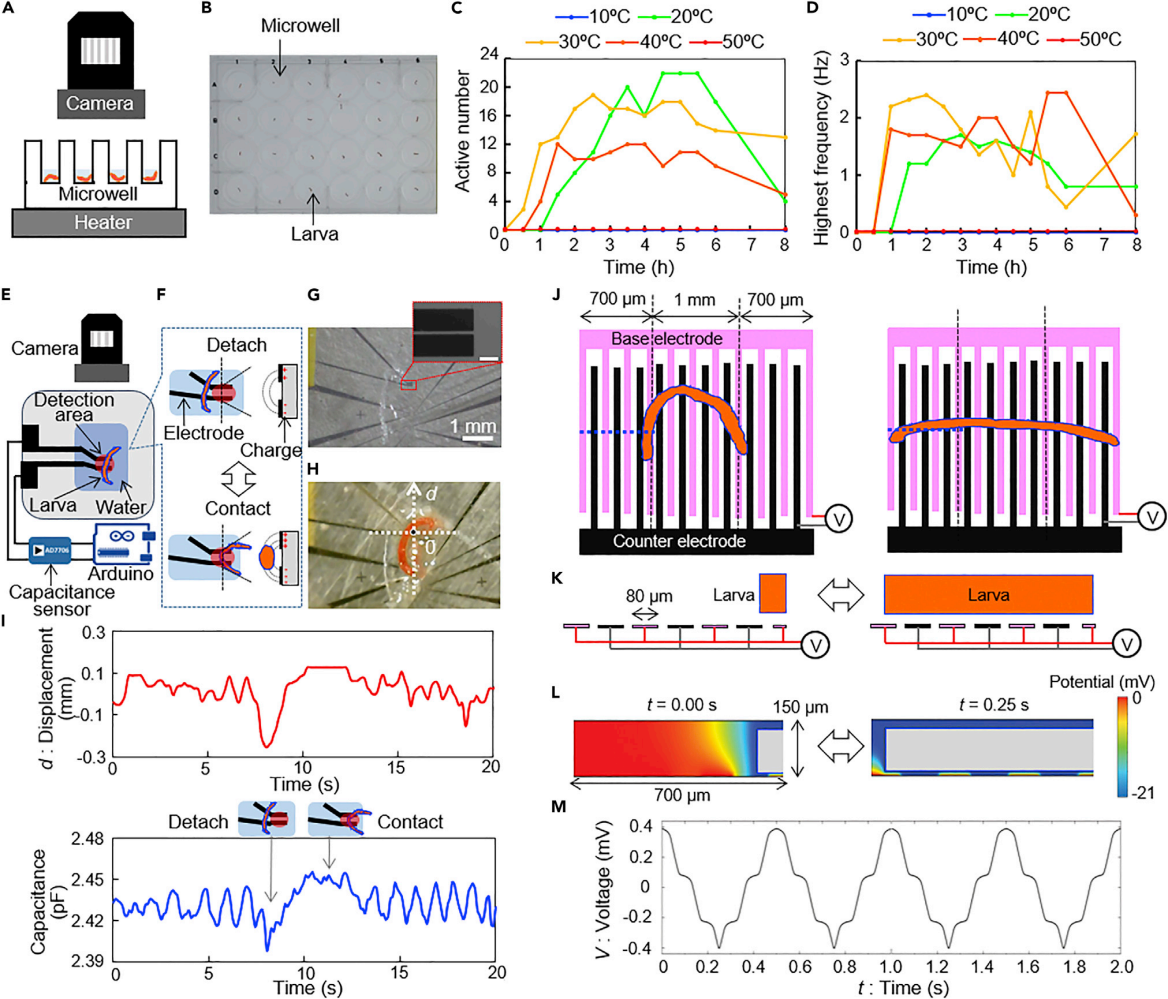
Property investigation of the larvae (A) Experimental set-up to investigate the motion of the larvae at various temperatures. (B) Photo of the larvae individually contained in a 24-microwell plate. (C) Time-course for the active number among a group of 24 larvae at various temperatures. (D) Time-course for the highest frequency among a group of 24 larvae at various temperatures. (E) A parallel electrode device and experimental set-up to investigate the electrical property of the larva. (F) Enlarged (left) top and (right) cross-sectional images around the larva along with the broken lines of top figures. Capacitance change can be measured by the transformation of electric field (dash curves) caused by the motion of the larva. (G) Photo showing the measurement area. Inset: a magnified image of the electrode edge forming the parallel detection area (scale bar: 50 μm). (H) Photo showing a larva on the parallel electrode device. The coordinate indicates the traced point on the outer body line of the larva (0) and the displacement of the point (*d*). (I) Time course for the displacement (*d*) and capacitance of the larva on the electrode. The times of the two graphs are synchronized. (J) Top and (K) cross-sectional view along the blue broken lines in (J) of the simulation model for power generation using the larva (left, shrinking; right, stretched state of the larva). (L) Simulation results of electric potential around the larva and electrodes. Gray color indicates the inside of the larva. (M) Simulated time-course of generated voltage by the larva moving at 2 Hz frequency.

In this experiment, the fundamental properties of the chironomid larvae were analyzed prior to specifically designing the microdevice. The recovery rate and the motion of the larvae were visually monitored when they were recovered in a 24-microwell tissue culture plate filled with Milli-Q water (1.5 mL/microwell). A dried larva was placed in each well after the water temperature was stabilized and the motions of each larva were monitored and recorded by a camera every 30 min for 8 h (Figures 2A and 2B). After being placed in the water, the dark red dried larvae gradually expanded and became bright red or white. To observe the response of the larvae at different temperatures, the experimental temperatures were changed in 10°C steps from 10°C to 50°C.

As shown in Video S1, there were large individual differences, but some of the larvae began to move with a bending-stretching motion of 1–2 Hz frequency. The required time for at least one larva in 24 wells to recover (that is, to begin moving) is summarized in Figure 2C. The larvae gradually recovered within 3 h for temperatures of 20, 30, and 40°C. The largest ratio and also the shortest recovery time were observed at 30°C. The highest motion frequency measured among the group of 24 larvae is plotted in Figure 2D. The graph was similar to the plots for the number of recovered larvae. These results indicated that a certain number of larvae was required to get the overall behavior of larvae for the same set of conditions. Also, both the recovery number and the highest frequency among the recovered number of larvae were found to be correlated with the activity of the larvae.

Video S1. The motion of chironomid larvae, related to Figure 2 This video shows the motion of chironomid larvae in a 24-microwell plate was observed 2 h after the larvae were placed on the plate at 30ºC.

### Investigation of the motion and electrical property of a single chironomid larva

To specifically design the vibration-based power generation device, the activity of single chironomid larva was analyzed in detail. Although the averaging motion was analyzed in the previous section, it is indispensable to use single larva for the detailed investigation of activity such as the electrical capacitance change and dynamical displacement change. To measure the motion of a larva, an actively moving chironomid was selected and put on a glass slide. A USB zoom video camera connected to a PC was prepared to monitor the motion of the larva and the desktop images were recorded. The image data were binarized as white-black, and the y axis displacement of the outer body line of the larva was monitored.

Moreover, to prove that the larva has an electrical charge in water for power generation, capacitance was measured simultaneously using a pair of parallel electrodes (Figures 2E–2H). These parallel electrodes were made of Pt and fabricated by a lift-off process (Moriguchi et al., 2015) (details are given in method details and Figure S1). The electrode edge was the detection area to measure the capacitance between the electrodes. When a larva was in the detection area, the capacitance was changed owing to the difference in the relative permittivity of water and the larva. To amplify the signal, a constant voltage (2 V) was applied to the larva and the capacitance was continuously monitored at a 30 Hz sampling rate. The image of the larval motion and the wave data of the capacitance were displayed together, and the motion was analyzed using the video images (Video S2).

Video S2. The capacitance measurement, related to Figure 2 This video shows the real-time capacitance measurement result (left) with the synchronized camera image of a chironomid larva on the parallel electrode device (right) 2 h after putting the larva on this device for observation at 30ºC.

The actual displacement of the larva repeatedly bending and stretching depended on the measurement part but from the image data, we assumed measurement for an area of a few hundred micrometers (Figure 2H). The capacitance was about 2 pF and there was a clear difference compared to water alone. This meant that the larva had a surface charge when it was actively moving.

As input for the simulation described in the next sub-section, we needed to measure the natural surface charge of the larva in water experimentally. The charge of an active larva when it was just immersed in water was obtained as follows. The surface potential of the larva was measured as described in method details and we estimated it as −21 mV. Although this is not for a living larva, it can be used as a reference value for the simulation.

This experiment also demonstrated that the motion of the larva could be electrically detected by the microdevice. By continuously measuring the capacitance, it can be judged whether the larva is in the detection area or not just as reported for droplet detection previously (Elbuken et al., 2011). The image data were well synchronized with the capacitance change as shown in Figure 2I. Even though this microdevice used electricity to amplify the signal, it was confirmed that motion capturing of a larva is possible by using MEMS-based electrodes.

### Simulation of the power generation by the motion of the chironomid larva

Even though the motion of the chironomid larva was detected by the microdevice in the previous section, the detection area was so small that the larva had to be precisely positioned on the detection spot. Furthermore, we selected the most active larva this time, but actually the individual motions were very different and a part of them was not recovered. These issues prevent application to environmental sensing in addition to the requirement of the external power source. To address them, a microdevice with a large detection area and a simultaneous detection system using a number of larvae with power-free sensing exploiting the vibration-based power generation must be developed. For this purpose, interdigital comb electrodes were employed as explained in the introduction.

We roughly estimated the generation power of the microdevice with a chironomid larva by simulation. Based on the measured surface potential of the larva (−21 mV), power generation performance by the motion of a larva was calculated.

COMSOL simulation software was used to consider the situation with a larva moving on multiple-electrode comb teeth (80 μm wide and 20 μm gap width between the teeth). As observed in previous sub-sections, the larva repeated bending and stretching motions. From a cross-sectional viewpoint, the length of the larva was changing owing to the motion of the larva, which caused the variation in the number of teeth of the interdigital comb electrodes under the larva. The actual length of a larva is 1–2 mm which exceeds the simulation area of this software. Therefore, in simulation, the larva was simplified as a 2D square (80–600 μm length, 100 μm height) in pure water. The simulation area (680 × 130 μm) was assumed to be symmetrical, and one side contains 6.5 electrodes. During simulation, the length of the larvae varied between 80 and 600 μm with a speed of 2.08 mm/s, so that to simulate the vibration in the number of covering electrodes during the larval movement. The moving speed of the larva was determined based on the frequency of detected signals (2 Hz) from the estimation in the previous sub-sections.

The potential of one side of the pair of electrodes was set to 0 V, leading to the current signal flowing from one electrode to the other electrode. When the larva moved over the electrodes (a distance of 10 μm), a different electric field distribution resulted between the larva and the electrodes, and a different current signal also resulted between the electrodes as shown in Figures 2J–2L and S2. The simulated current signal was transferred to the voltage signal using the measured microdevice resistance value (150 Ω) as in experiments.

When the number of teeth of the comb electrodes under the larva changed, the output voltage signal fluctuated around 0 V in the waveform (Figure 2M). As all parameters used in the simulation were based on real experiments, the amplitude of the simulation result (about 0.8 mV) could be used as a reference for the real experimental data. Likewise, the simulation showed that the motion of the larva was enough to generate a detectable voltage when using the interdigital comb electrodes.

Additionally, simulations of “40 μm electrode width and 10 μm span” and “160 μm electrode width and 40 μm span” designs are also indicated in Figure S3. It is shown that the power increases with higher integration. Although the power can be increased by using a more integrated one, the higher integrated device has a risk of disconnection or insulation breakdown. Therefore, we chose the design in Figure 2.

### Demonstration of power generation using a microdevice with interdigital comb electrodes

**Figure 3:**
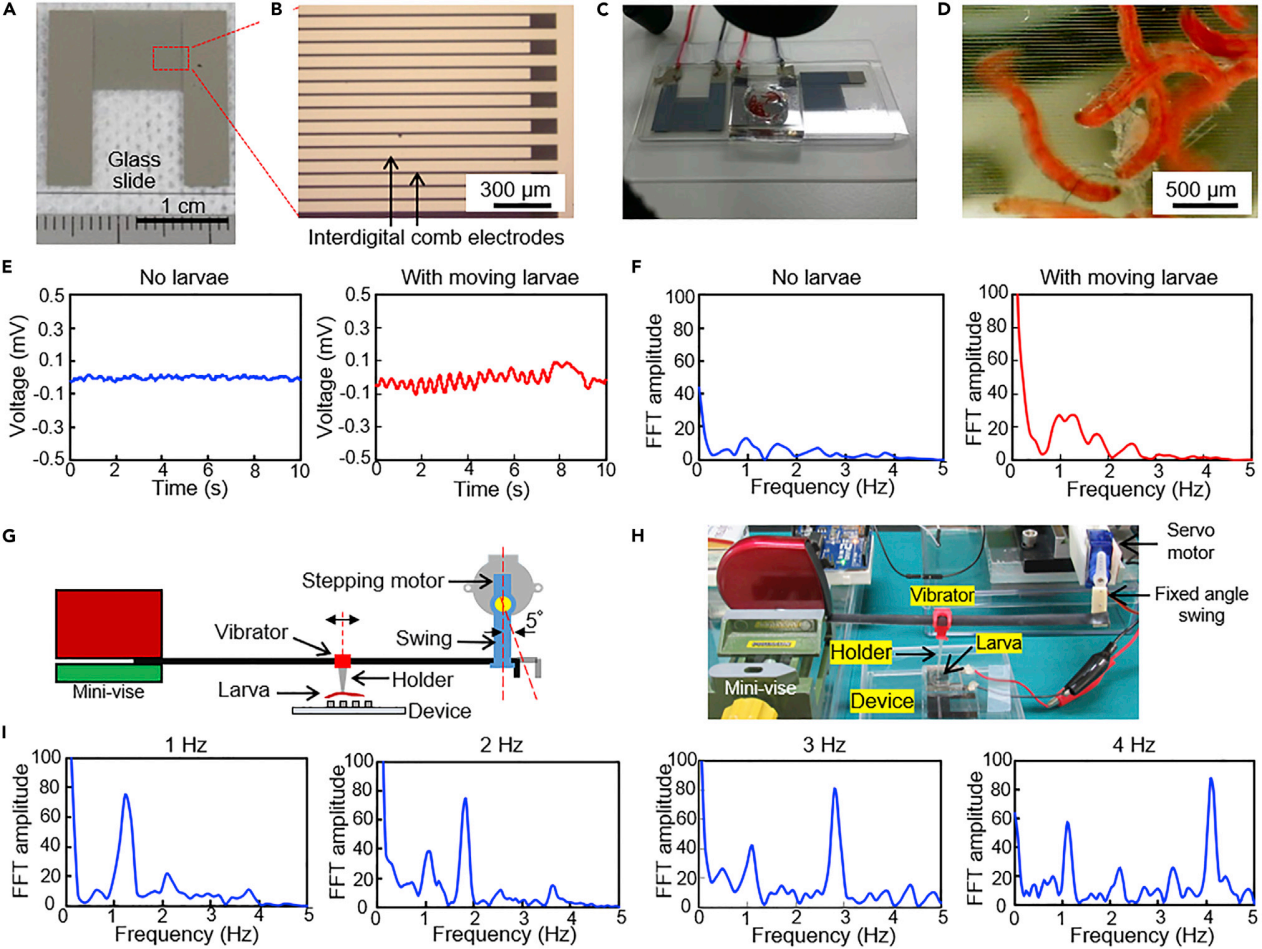
Demonstration of power-free biosensing using larval motion (A) Photo of the microdevice with a pair of interdigital comb electrodes to detect the motion of the larvae. (B) Photo of an interdigital comb electrode. (C) Photo of the device showing the larvae and PDMS pool filled with water. (D) Photo of the moving larvae on the electrodes. (E) Time-courses for the generated voltage of the conditions of “no larvae” and “with moving larvae at 30°C, 1 h after being immersed in water.” (F) Fast Fourier transform (FFT) amplitude versus frequency of the conditions of “no larvae” and “with moving larvae at 30°C, 1 h after being immersed in water.” (G) Schematic drawing of the experimental set-up to detect the constant vibrating motion of a larva. (H) Photo of the set-up. (I) FFT amplitudes for the conditions providing 1, 2, 3, and 4 Hz frequencies using the constant vibration machine.

Sensing of the larval motion was demonstrated based on the power generation by the vibration using the microdevice with interdigital comb electrodes. These electrodes were also fabricated by the lift-off processing; 60 comb teeth of the Ni electrodes, with 80 μm comb tooth width (the gap width between the teeth was 20 μm) were fabricated on a glass slide (30 × 70 mm, thickness: 0.7 mm). The detailed process is described in method details and Figure S1. Photos of the fabricated microdevice are shown in Figures 3A and 3B. To confine the larvae in the electrode area, a PDMS pool was put on the microdevice. The dry larvae were kept in the pool and pure water was added so that the larvae absorbed it and recovered. The interdigital comb electrodes were connected with the data logger to monitor and record the generated voltage when the larvae started to move. To compare the obtained electrical data with the actual movement of the larvae, a USB zoom camera was used to record their movement. Temperature was controlled by the temperature regulation plate.

The chironomid larval motion was optically confirmed on the microdevice (Figures 3C, 3D, Videos S3 and S4). The data were acquired at 30°C, 1 h after applying water. These were the conditions under which the larvae were the most active as described earlier. In this condition, eight of 10 larvae became active which is similar to that using the well plate experiment. From the measurement of the generated voltage, waves around 2 Hz frequency were found for the case of moving larvae but not with the control (no moving larvae) (Figure 3E). The amplitude of the voltage was about 0.11 mV and the corresponding current was estimated as 730 nA based on the measured resistance of the device (150 Ω), which was smaller than the simulation result. This was probably because the distance from the electrode to the larva was longer than 10 μm when it was moving rapidly. However, the order of the generated power was the same.

Video S3. The voltage measurement (without motion), related to Figure 3 This video shows the real-time voltage measurement result (left) with the synchronized camera image of chironomid larvae on the vibration-based power generation device (right) just after putting larvae on this device for observation at 30ºC.

Video S4. The voltage measurement (with motion), related to Figure 3 This video shows the real-time voltage measurement result (left) with the synchronized camera image of chironomid larvae on the vibration-based power generation device (right) 2 h after putting larvae on this device for observation at 30ºC.

However, just by these data, it was difficult to judge that the motion of a larva was captured. Therefore, fast Fourier transform (FFT) processing was applied to the data (Huang et al., 2019) as described in quantification and statistical analysis. A clear peak was detected around 1–2 Hz in the FFT curve compared with the control data (Figure 3F). This peak well represented the unique motion of these larvae.

### Validation of the device using a constant vibration machine

To verify the detection ability of the microdevice, a mechanically controlled constant vibration machine was used. This constant vibration machine converted constant oscillations of the servo motor to reciprocating linear movements of the larva. It replaced the natural biological motion of the larva with the fixed frequency motion of the vibrator (swing-vibration).

The vibrator was fixed above the microdevice, and the Arduino Uno microcontroller was used to make the larva on the vibrator move repeatedly at a fixed frequency (Figures 3G, 3H, and Video S5). Electrical signals at the frequency of 1–4 Hz with 1 Hz steps were measured (Figure S4) and analyzed to detect the constant frequency wave caused by the machine. The acquired amplitude curve after performing the FFT processing is shown in Figure 3I. Clear peaks at the corresponding frequency were detected. Although there were some small peaks around 1 Hz at 2, 3, and 4 Hz constant vibration conditions, these were unavoidable sub-peaks included in FFT analysis data. Therefore, the ability of the device to detect the frequency of the larval motion and the data reliability were proved.

Video S5. The working constant vibration machine, related to Figure 3 This video shows the actuation of the constant vibration machine used to verify the detection ability of the microdevice. The oscillation frequency of the servo motor stick was approximately 3 Hz in the video.

### Investigation of temperature dependency

**Figure 4:**
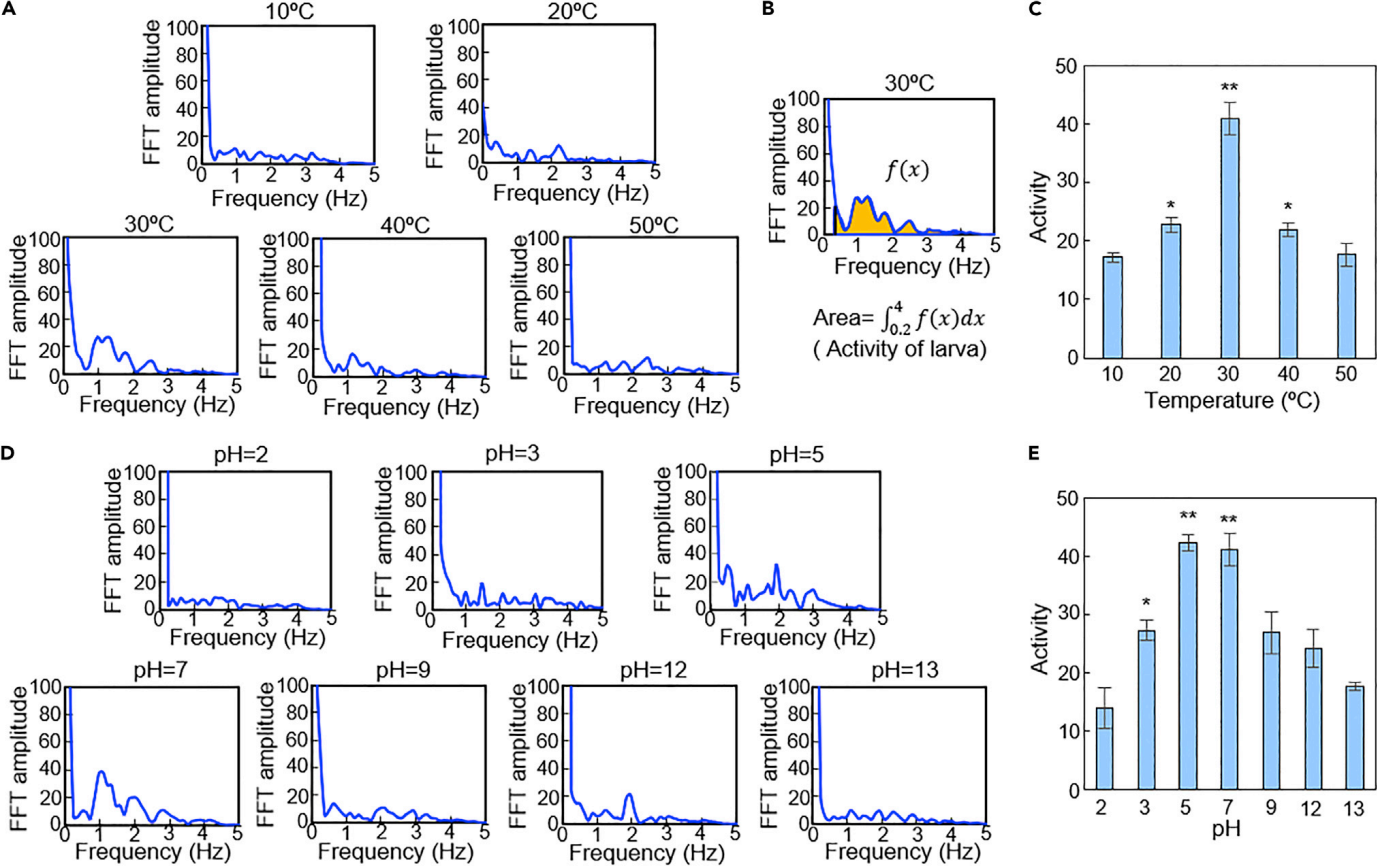
Demonstration of power-free bio-sensing of temperature and pH using the microdevice (A) FFT amplitudes versus frequency at temperatures of 10, 20, 30, 40, and 50°C. (B) Estimation of “activity” from the FFT amplitude versus frequency graph. (C) A graph of estimated activity versus temperature. (D) FFT amplitudes versus frequency at pH values of 2, 3, 5, 7, 9, 12, and 13. (E) A graph of estimated activity versus pH. Data of (C) and (E) are represented as mean ± SEM (n = 3). Symbols: ∗p < 0.05; ∗∗p < 0.01 compared with controls (condition of 10°C for (C) and pH = 2 for (E) with no active chironomid larvae); ∗p < 0.05, ∗∗p < 0.01 (Student’s *t* test).

The temperature dependency of the larval motion was demonstrated to be detected by the device. To confirm the sensing range of temperature, the temperature was set from 10 to 50°C with 10°C steps just as roughly measured in Figures 2C and 2D from the image data. From the FFT data at each temperature 2 h after applying water (Figure 4A), we found that the overall values of the curve were the highest at 30°C. However, to evaluate the activity of the larval motion more quantitatively, an index to define the activity is required. As discussed in the image analysis experiment, both the recovery number and the frequency correlated with the activity of the larvae. In FFT graphs, the recovery number vibrating at the specific frequency (*x*-axis) roughly indicates the FFT value (*y* axis). Therefore, the integration value (area) in a specific frequency range can be assumed to represent the activity of the larvae. Here, as shown in Figure 4B, the FFT amplitude values under 0.2 Hz were removed because of the large background owing to artifacts. On the other hand, the upper limit of the larvae activity looked smaller than 4 Hz from the video data. Then, a 0.2 Hz high-pass filter and 4 Hz low-pass filter were applied to the acquired signals to separate signal fluctuations caused by the biological motion of the larvae. The integration of the FFT amplitude values in a 0.2–4 Hz bandwidth was defined as activity. This approach is known to minimize the effect of readout noise at high frequency (Chawla et al., 2018).

The activities of the larvae at each temperature are plotted in Figure 4C. Clear peaks could be seen at 30°C. Moreover, the intermediate activity of larvae at 40°C could also be detected. At 20–40°C, significant differences were confirmed compared with 10°C with no active chironomid larvae. We demonstrated that the activities could be monitored by the microdevice, and also that it could be used for temperature sensing.

### Investigation of pH dependency

This microdevice could be used for sensing not only physical parameters but also chemical parameters. To prove this, the pH dependency of the signal in a large range was investigated. Different buffer solutions are used for pH ranges because their buffer capacity is limited. In this experiment, differences in salts for each buffer solution should be eliminated in order to investigate the effect of pH on the larvae. Therefore, a buffer solution with a wide range of buffering capacity was used for both acid and alkali solutions (method details). The temperature was constant at 30°C and the data were obtained 2 h after starting the experiment.

FFT graphs at pH from 2 to 13 are shown in Figure 4D. From the activity index data shown in Figure 4E, we saw a peak around pH = 5–7. At least in pH = 3–7, significant differences were confirmed compared with pH = 2 with no active chironomid larvae. Also, we found that the larvae had stronger resistance to an acid environment than to a base one. We judged the microdevice was applicable to pH sensing.

### Investigation of heat-resistance of larvae in vacuum conditions

Additionally, we confirmed the recovery of larvae after continuous exposure to heat under vacuum conditions to verify that our device can be used in outer space. The details are described in supplementary Text and Figure S5.

## Discussion

Although the electricity is used to monitor the generated power, the core part of the sensing is power-free owing to the autonomous power generation. This is the most important characteristic of the demonstrated microdevice if it is to be used for the exploration of extreme environments.

A typical low power consumption sensing system usually consists of three parts: sensing, signal amplification, and A/D analysis and signal transmission. MEMS sensors have been reported for temperature (Veeramani et al., 2018) and pH (Zhang et al., 2017) which serve as precedents for our microdevice, although these sensors can monitor just one parameter. Such low power consumption sensing components still consume over a few hundred nanoamperes per each type (and at least three types of sensors are required), which are the largest loads in a complete system. On the other hand, in our proposed microdevice with the interdigital electrodes, multiple sensing components (larvae) actively generated electrical signals up to 0.11 mV and 730 nA without an external power supply.

The obtained power (80 pW) is small as an energy harvesting device, but it is useful for “wake up” power of the communication device and has the potential to significantly increase the lifetime and efficiency of outer space exploration systems. Although the generated power is not enough to drive devices such as a data logger, an analysis system, or communication devices, it is enough for a trigger to drive devices that are usually in a stand-by status. For example, 0.11 mV can be amplified to 1.6–2.9 V by a pre-amplifier such as the TSU101 manufactured by STMicroelectronics (1.65 V, 600 nA) (STMicroelectronics Datasheet). Such amplified signals could be used as a wake-up signal to activate a commercial ultra-low power consumption microcontroller such as the MSP430F20, manufactured by Texas Instruments (1.65 V, 600 nA), from its deep sleep mode (Texas Instruments Datasheet). In such an arrangement, the wake-up signal could be used to start A/D data collection, analysis, making judgments, and transmitting signals when the larvae start to move. Moreover, it is important that the proposed device does not need an external power source for sensing. As shown in Table 1, conventional commercially available sensors consume power to maintain the basic functions. In case of measuring various factors for the evaluation of the habitable environment, more power is necessary. On the other hand, the proposed live-sensing component is power free and has the potential to significantly increase the lifetime and efficiency of outer space exploration systems. In typical cases, a single power button battery (CR2032, 3V, 200 mAh) could support running of such an arrangement with full function for about 12 years. If the integration of the microcontroller and the pre-amplifier is customized, the lifetime of the system could be extended to a few decades. In summary, the live-sensing components increase the possibility to find target environments among outer space planets.

**Table 1:** Comparison of features between conventional sensors and proposed live-sensing device

Sensors	Detecting target	Current consumption	Function of energy harvesting	Capable of awaking microprocessor
STS40 (Sensirion)	Temperature	0.4 μA	No	No
SHTC3 (Mouser Electronics)	Humidity	0.5 μA	No	No
FDO2 (PyroScience)	Gas (O_2_)	8000 μA	No	No
ICP-10100 (TDK)	Pressure	1.3 μA	No	No
Device created in this research	Total habitable environments: gas, air pressure, temperature, humidity	0 A	Yes, 0.7 μA	Yes, >0.6 μA

We also compare our microdevice with conventional microdevices from the viewpoint of exploiting the function of biological materials to confirm the uniqueness of our device. Microfluidic devices are widely used for biochemical sensing using cells or biomolecules. For instance, there have been reports of molecular sensors using cell membrane proteins (Misawa et al., 2010, 2019; Yamada et al., 2021), and cancer detection sensors using antibodies (Chikkaveeraiah et al., 2012) or using microorganisms (Su et al., 2011; Melkmana and Sengupta, 2004). Although these sensors have high sensitivity, they can detect just one parameter. From another viewpoint, our device can also be regarded as a life-machine fusion device exploiting the activating function of life systems on a microdevice (Lin et al., 2021; Feinberg, 2015). There have been reports of actuators using microorganisms (Hiratsuka et al., 2006; Weibel et al., 2005), cultured muscle tissues (Park et al., 2016; Raman et al., 2016; Nawroth et al., 2012; Tanaka et al., 2008), or organs (Tanaka et al., 2016, 2017; Shoji et al., 2016). However, these devices fundamentally use a single function of the cells, tissue, or organs. On the other hand, our device uses whole multi-cell individuals. As such, this device is unique in the use of multi-functions of the whole living individual.

In this study, we demonstrated a multi-sensing MEMS-based device using the motion of larvae. This device exploits various sensing performances of a living system which are expressed as “motion.” By detecting motion as one parameter (electrical signals), environmental sensing is possible without arbitrariness.

As this device exploits a biological system, it can be used in searching for a survivable environment. It can be used not only in outer space, but everywhere to simply detect water, temperature, and chemical states of the environment simultaneously. Even on Earth, there is interest in searching for habitable zones in wildernesses, deserts, or polar regions. Following testing in these Earth regions, our device will be applied to more distant and extreme environments like on other planets.

### Limitations of the study

The current device can detect only one parameter in one experiment. As a future issue, various parameters must be detected simultaneously. For this purpose, more data are necessary, and the machine learning method would be useful. Furthermore, the sensitivity should be increased. Ni electrode used in this experiment is also usable for the magnetically induced power generation to generate higher electricity by combining magnetic materials with chironomid larvae. By addressing such issues, our device will become a more practical biohybrid device for exploration.

## STAR★Methods

### Key resources table

**Table:** 

REAGENT or RESOURCE	SOURCE	IDENTIFIER
**Biological samples**		

*Polypedilum vanderplanki* (larva)	Watanabe et al., 2002	N/A
Chemicals		
Sodium chloride	Sigma-Aldrich	CAS: 7647-14-5
hexamethyldisilazane	Sigma-Aldrich	CAS: 999-97-3
Positive photoresist	Merck	AZ1500 38 cp
Developer	Merck	AZ developer
Phosphoric acid	Nacalai Tesque	CAS: 7664-38-2
Boric acid	Nacalai Tesque	CAS: 10043-35-3
Acetic acid	Nacalai Tesque	CAS: 64-19-7
Sodium hydroxide	Fujifilm Wako Pure Chemical	CAS: 1310-73-2

**Software and algorithms**		

COMSOL Multiphysics	COMSOL	ver. 5.6
Bandicam	Bandicam Company	N/A
Windows Movie maker	Microsoft	ver. 2012
Kinovea	Kinovea Computer Software	ver. 0.8.27
WAVE LOGGER PRO	Keyence	ver. R4.02.00
Excel	Microsoft	ver. 2021

### Resource availability

#### Lead contact

Further information and requests for resources and reagents should be directed to and will be fulfilled by the lead contact, Yo Tanaka (yotanaka1980@gmail.com).

#### Materials availability

This study did not generate new unique reagents.

### Experimental model and subject details

#### Chironomid larvae

*Larvae of the African chironomid, Polypedilum vanderplanki* (*P. vanderplanki*) were reared on a 1% agar diet, containing 2% commercial milk, under controlled conditions (13 h light followed by11 h dark; temperature 27 to 28°C) according to the established protocol (Watanabe et al., 2002). The induction of anhydrobiosis by desiccation has been described previously (Watanabe et al., 2003). The desiccated larvae were stored in dry, light-shielded, airtight packages at room temperature. All experiments were performed in Institute of Agrobiological Sciences, NARO, Japan.

### Method details

#### Measurement of surface charge of chironomid larvae for the simulation

The surface charge (zeta-potential) was measured by laser doppler electrophoresis with an electrophoretic light scattering apparatus (ELSZ-2000Z, Otsuka Electronics). When electrical fields are applied to micro-object dispersion, the objects migrate in oppositely charged directions. When the objects are irradiated during migration, scattered light causes the Doppler shift which depends on electrophoretic mobility (laser Doppler method) (Clogston and Patri, 2010).

To apply this method, the objects to be measured should be crushed into small pieces. Therefore, 10 dried larvae with a total weight of 1.7 mg were randomly selected for the potentiometric measurement. The selected larvae were put into a mortar and ground with a pestle until 70% of the ground particles were smaller than 5 μm. Finally, the ground particles were dissolved into 5 mL of saline (10 mM) (S5886-500G, Sigma-Aldrich).

All simulations were made using COMSOL Multiphysics 5.6 (COMSOL Inc.).

#### Fabrication of a microdevice with Ni or Pt electrodes

First, a glass slide was cleaned by immersing it in H_2_SO_4_/H_2_O_2_ solution for 15 min before treating it in a plasma etching system (FA-1, Samco) at 30 W for 2 min. Then, the slide was spin-coated with HDMS (hexamethyldisilazane, Sigma-Aldrich) at 500 rpm for 5 s followed by 5000 rpm for 40 s with a spin-coater (1H-D7, Mikasa) to enhance the adhesion of the slide surface. The coated slide was baked at 80°C for 5 min using a hotplate (Ninos ND01, As one). After that, positive photoresist (AZ1500 38 cp, Merck) was coated using the same conditions, followed by baking at 80°C for 10 min on the hotplate. The baked slide was exposed to UV light (405 nm, 150 mJ/cm^2^) using a maskless lithography system (DL-1000, Nano System Solutions) to make the patterns of the electrodes before development using AZ developer (Merck). A Ni or Pt film (100 nm thick) was sputter-deposited on the patterned slide by a sputtering machine (EIS-220, Elionix). The slide was immersed in acetone for 12 h to remove the unexposed region and excess Ni or Pt metal. SiO_2_ was then deposited as an isolation layer (100 nm thick). The slide substrate with patterned electrodes was rinsed in pure water and blow-dried using N_2_ flow. Finally, wires were connected using electrically conductive glue.

#### Fabrication of the constant vibration machine

The main components of the constant vibration machine included: a single-board Arduino Uno microcontroller (SMD R3, Elegoo), a servo motor (Micro servo SG90, Elegoo), a mini-vise (Proxxon, Föhren) and a spring tape. The oscillation code of swing-vibration motion was uploaded onto the Arduino Uno microcontroller and it was wired to the servo motor. The motor oscillating angle was set between 0 to 5°. Then, the servo motor and the spring tape were fixed in place. The chironomid larva was placed in a holder and the holder was placed onto the spring tape. The maximum horizontal linear moving range was 2.81 ± 0.18 mm (n = 3, the error is ±SD).

#### Image capture and data analysis

For the image analysis, a commercially available 24-microwell plate (TPP Techno Plastic Products AG) and Peltier-based temperature controller (VPE-35, Vics) were used. A digital camera (D810a, Nikon) and 20 cm focus distance lens (AF 18200mm, Tamron) was directly positioned above the tissue culture plate. For microscale observations on the microdevice, a USB video camera (30 fps, DANIU, Banggood) was used. The desktop images were recorded using Bandicam (Bandicam Company). The recorded video data were edge-enhanced and binarized by Windows Movie maker (vers. 2012, Microsoft), and the displacement of the chironomid larva was measured by Kinovea (vers. 0.8.27, Kinovea Computer software) from the edited video data.

#### Preparation of buffer solutions of different pH

Britton–Robinson buffer was used as a universal buffer and has a buffering capacity from pH 2 to 12 (Britton and Robinson, 1931). First, an acid solution and an alkaline solution were prepared. The acid solution was a mixture of phosphoric acid (27618-55, Nacalai Tesque), boric acid (05215-05, Nacalai Tesque), and acetic acid (00212-85, Nacalai Tesque). The concentration of each acid after mixing was 40 mM. The alkaline solution was a 200-mM sodium hydroxide (190–14565, Fujifilm Wako Pure Chemical) solution. The acid and alkaline solutions were mixed while being measured with a pH meter (D-71, Horiba Advanced Techno) to prepare buffer solutions of pH 2, 3, 4, 5, 6, 9, 10, 11, and 12. For pH = 7, the data using the pure water was used. Each buffer solution was diluted 10-fold with water at the time of use.

#### Electrical measurement

The viability of larvae was measured with an electrical signal collector every 30 min for 8 h. To obtain the DC voltage signal (waveform data) with the microdevice, a data logger (NR2000, Keyence) with a high-speed analog measurement system (NR-HA08, Keyence) and software (WAVE LOGGER PRO, Keyence) was used with a sampling rate of 1 kHz.

### Quantification and statistical analysis

#### Data analysis of electrical measurement

The obtained waveform data by electrical measurement were divided into several small segments with a length of 10 s, and converted to csv files. The files were opened with spreadsheet software (Excel 2021, Microsoft) and moving average was calculated within the interval of 200 points. After that, the data were processed by FFT using a Hamming window as the window function and 4096 sample points.

#### Data analysis for activity evaluation of the larvae

The activity of the larvae was defined as the integration of the FFT amplitude values in a 0.2–4 Hz bandwidth as described in the main text. The values of activity were plotted in each temperature or pH condition and compared with the control data (at 10°C or pH = 2). Data were compared across conditions with Student’s t tests, and p values < 0.05 were regarded as significant. The values were calculated with the same software used in the previous section (Excel 2021). Statistical details are described in the legend of Figure 4.

## Data Availability

• All data reported in this paper will be shared by the lead contact upon request. • This paper does not report original code. • Any additional information required to reanalyze the data reported in this paper is available from the lead contact upon request.
