# Supplementary material for: Anhydrobiotic chironomid larval motion-based multi-sensing microdevice for the exploration of survivable locations
Source: iScience. 2022 Jul 20;25(8):104639. doi: 10.1016/j.isci.2022.104639 (PMC9418600; doi:10.1016/j.isci.2022.104639)
Supplement: Document S1. Figures S1–S5 [file mmc1.pdf]

iScience, Volume *25*

Supplemental information

Anhydrobiotic chironomid larval motion-based multi-sensing microdevice for the exploration of survivable locations

Yo Tanaka, Doudou Ma, Satoshi Amaya, Yusufu Aishan, Yigang Shen, Shun-ichi Funano, Tao Tang, Yoichiroh Hosokawa, Oleg Gusev, Takashi Okuda, Takahiro Kikawada, and Yaxiaer Yalikun

Supporting Information

Anhydrobiotic chironomid larval motion-based multi-sensing microdevice for exploration of survivable locations

Yo Tanaka,[1,2]* Doudou Ma,[1,2] Satoshi Amaya,[1] Yusufu Aishan,[1,2] Yigang Shen,[1] Shun-ichi Funano,[1] Tao Tang,[3] Yoichiroh Hosokawa,[3] Oleg Gusev,[4] Takashi Okuda,[5] Takahiro Kikawada,[5] Yaxiaer Yalikun[1,3]

[1] Center for Biosystems Dynamics Research (BDR), RIKEN, 1-3 Yamadaoka, Suita, Osaka 565-0871, Japan

[2] Graduate School of Frontier Biosciences, Osaka University, 1-3 Yamadaoka, Suita, Osaka 565-0871, Japan

[3] Graduate School of Nara Institute of Science and Technology, 8916-5 Takayamacho, Ikoma, Nara 630-0192, Japan

[4] RIKEN Cluster for Science, Technology and Innovation Hub (RCSTI), RIKEN, 1-7-22 Suehiro-cho, Tsurumi-ku, Yokohama City, Kanagawa, 230-0045, Japan
(Present Address: Graduate School of Medicine, 2 Chome-1-1 Hongo, Juntendo University, Tokyo 113-8421, Japan)

[5] Institute of Agrobiological Sciences, NARO, 1-2 Owashi, Tsukuba, Ibaraki 305-8634, Japan

*To whom correspondence may be addressed:

E-mail: yo.tanaka@riken.jp.

Table of Contents:

- **Supplementary Text**
- **Supplementary Figures (Figs. S1-S5)**

Supplementary Text

Investigation of heat-resistance of larvae in vacuum conditions.

Considering the application of the presented microdevice to use in outer space, it is indispensable to confirm the tolerance of the larvae to vacuum and heat conditions. There are large temperature fluctuations in outer space. For instance, the temperatures in the containers equipped on the ISS vary from -20 to 90°C (Novikova et al., 2011). Although temperature endurance has already been confirmed from -270 to 90°C (Hinton, 1960), continuous exposure of the anhydrobiotic larvae to temperatures exceeding 70˚C leads to a rapid decrease of the survival rate. The exposure time limit to such high temperatures under normal air circumstances was just a few minutes. In this case, it would be difficult to use our larvae-based biohybrid device for the space exploration. However, outer space is a vacuum, which is different from Earth.

In this experiment, to clarify the background of the larvae resistance to higher temperatures in outer space, we subjected the anhydrobiotic larvae to heat exposure under several atmospheric conditions (Fig. S5). We used a hydrolysis reaction tube to control the atmosphere. To expose the dried larvae to oxygen, oxygen gas was blown into the reaction tube from one open mouth, so that air could be pushed out from the other open mouth. The tubes were heated by a heat-block.

In agreement with previously reported data (Hinton, 1960), prolonged exposure of the anhydrobiotic larvae to heat under the normal air condition resulted in decreased survival. In contrast, the larvae exposed to heat in the vacuum condition showed no reduction in survival. Exposure of the larvae to heat under "oxygen-only" conditions resulted in

100% mortality.

This result provides evidence that the space vacuum acts as a thermo insulator, protecting the larvae from damage in outer space caused by overheating and reactive oxygen species. This thermo insulating phenomenon is helpful and important for long-term exploration use of our device.

Supplementary Figures and Legends

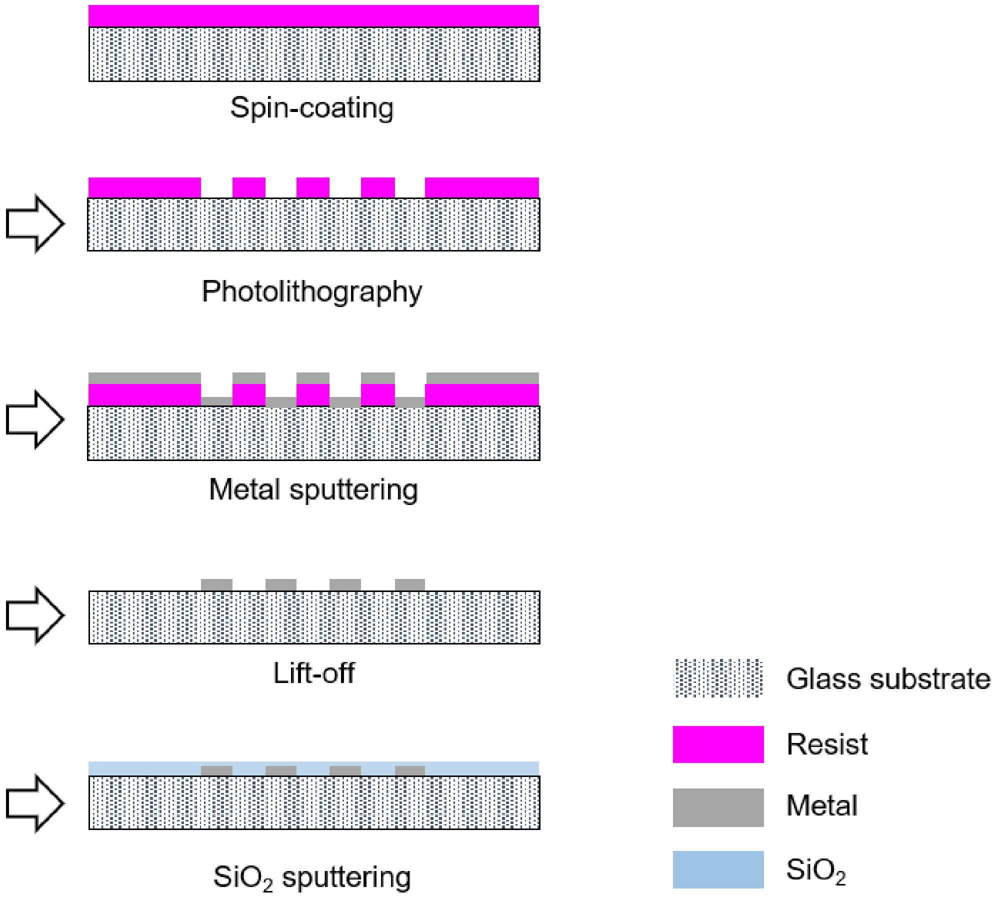

Figure S1 Fabrication process of the electrodes, Related to Figure 2. The parallel electrode device and interdigital electrode device were fabricated using mostly the same steps except for the metal used, Pt for the former and Ni for the latter.

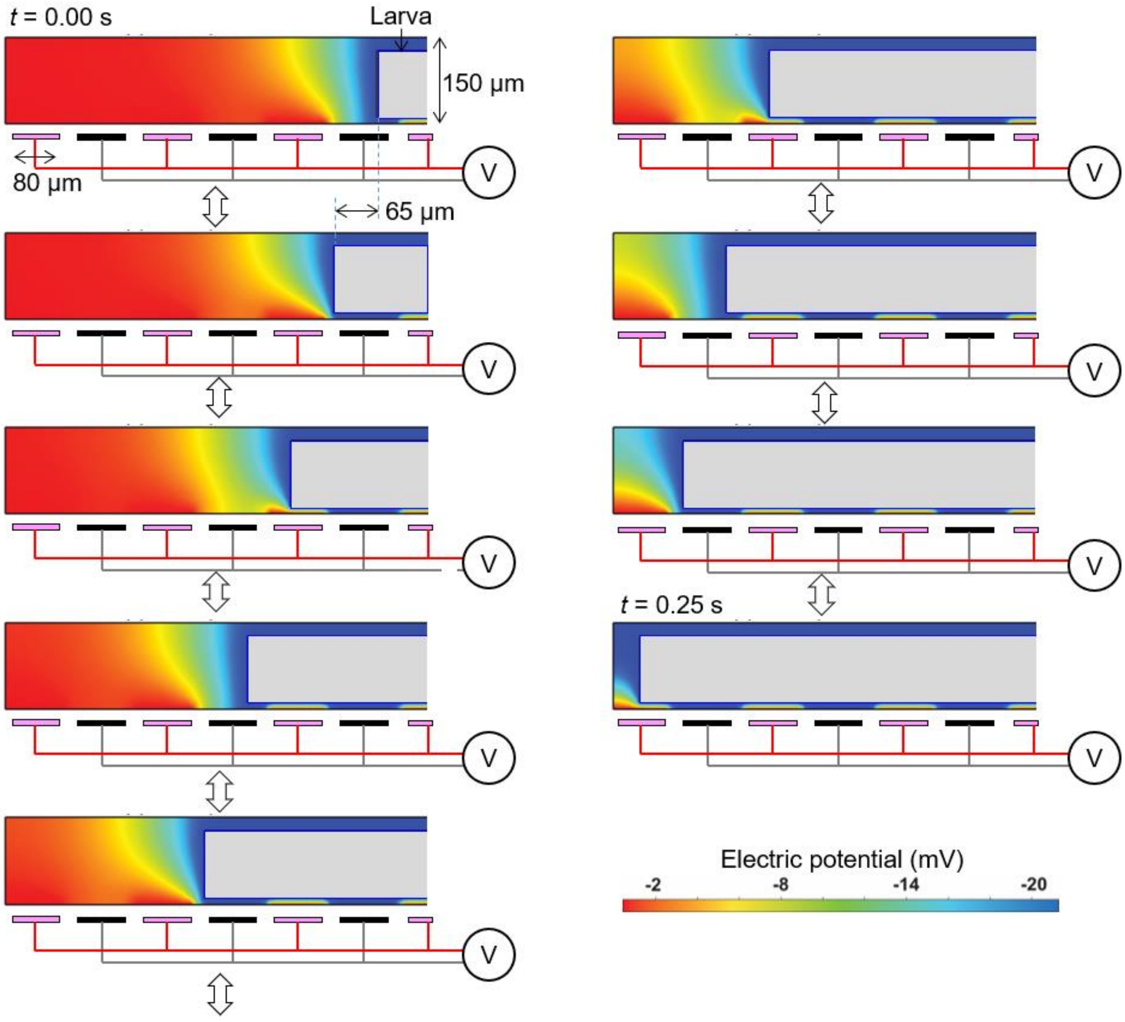

Figure S2 Extended simulation of power generation, Related to Figure 2. The simulation includes intermediate steps with 65 µm interval of elongation over the electrodes about the electric potential around the larva and electrodes shown in Figs. 2K-M. Grey color indicates the inside of the larva.

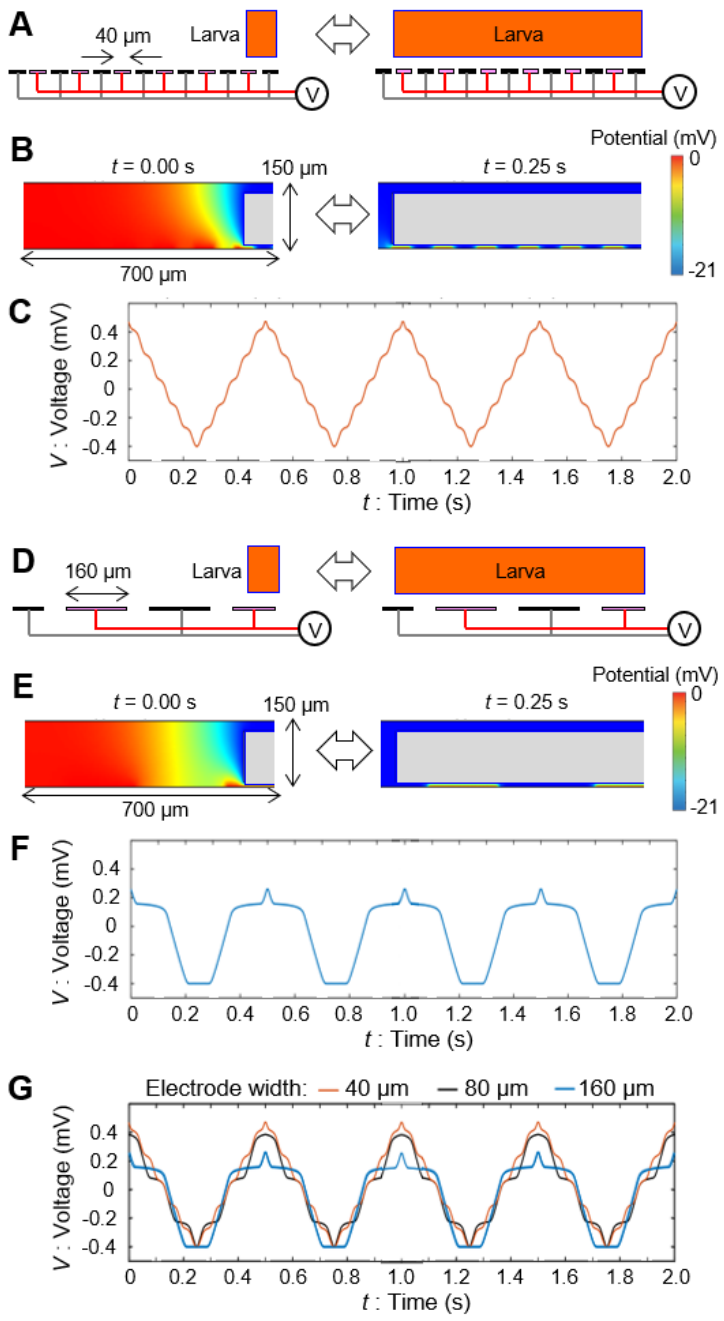

Figure S3 Additional simulation of power generation changing electrode width and span, Related to Figure 2. (A-C) "40 µm electrode width and 10 µm span" and (D-F) "160 µm electrode width and 40 µm span" designs corresponding to Figs 2K-M. (A,D) Simulation models. (B,E) Simulation results. (C,F) Simulated time-course graphs. (G) Overlaid time-course simulation graphs of all designs.

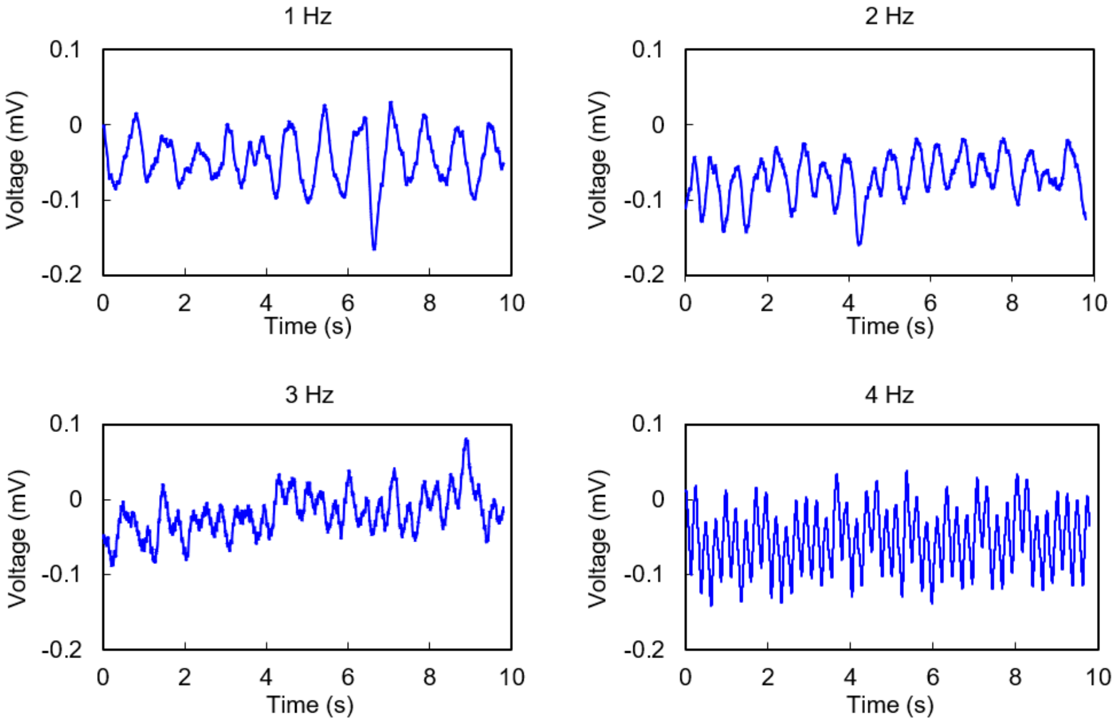

Figure S4 Raw data of the time-courses for the generated voltage, Related to Figure 3. The graphs show the results at the conditions of 1, 2, 3 and 4 Hz using the constant vibration machine.

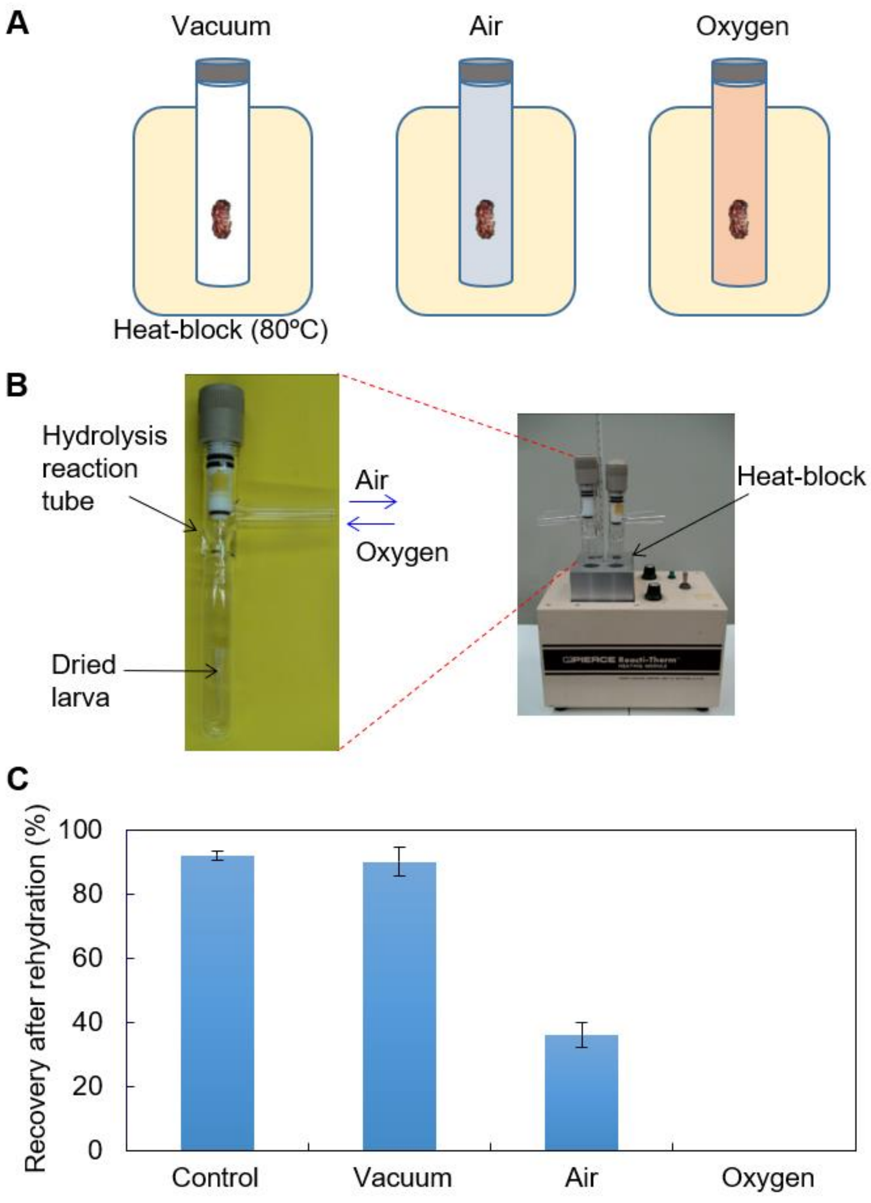

Figure S5 Measurement data for survival rate of the anhydrobiotic larvae after continuous exposure to heat under different atmospheric conditions, Related to Figure 3 and 4. (A) Experimental conditions. Larvae were exposed to 80°C for 20 h under vacuum, air, and oxygen. (B) Experimental set-up. (C) Recovery rate in each condition. "Control" refers to larvae kept at room temperature and in air. Data are represented as mean +/- SEM (n=3).
